# Functional hyper-IL-6 from vaccinia virus-colonized tumors triggers platelet formation and helps to alleviate toxicity of mitomycin C enhanced virus therapy

**DOI:** 10.1186/1479-5876-10-9

**Published:** 2012-01-11

**Authors:** Julia B Sturm, Michael Hess, Stephanie Weibel, Nanhai G Chen, Yong A Yu, Qian Zhang, Ulrike Donat, Cora Reiss, Stepan Gambaryan, Georg Krohne, Jochen Stritzker, Aladar A Szalay

**Affiliations:** 1 Department of Biochemistry, University of Würzburg, 97074 Würzburg, Germany; 2 Genelux Corporation, San Diego Science Center, San Diego, CA 92109, USA; 3 Department of Radiation Oncology, Rebecca & John Moores Comprehensive Cancer Center, University of California San Diego, La Jolla, CA 92093, USA; 4 Institute of Clinical Biochemistry and Pathobiochemistry, University of Würzburg, 97080 Würzburg, Germany; 5 Division of Electron Microscopy, University of Würzburg, 97074 Würzburg, Germany

**Keywords:** vaccinia virus, cancer, cytokine, hyper-IL-6, oncolysis, chemotherapy

## Abstract

**Background:**

Combination of oncolytic vaccinia virus therapy with conventional chemotherapy has shown promise for tumor therapy. However, side effects of chemotherapy including thrombocytopenia, still remain problematic.

**Methods:**

Here, we describe a novel approach to optimize combination therapy of oncolytic virus and chemotherapy utilizing virus-encoding hyper-IL-6, GLV-1h90, to reduce chemotherapy-associated side effects.

**Results:**

We showed that the hyper-IL-6 cytokine was successfully produced by GLV-1h90 and was functional both in cell culture as well as in tumor-bearing animals, in which the cytokine-producing vaccinia virus strain was well tolerated. When combined with the chemotherapeutic mitomycin C, the anti-tumor effect of the oncolytic virotherapy was significantly enhanced. Moreover, hyper-IL-6 expression greatly reduced the time interval during which the mice suffered from chemotherapy-induced thrombocytopenia.

**Conclusion:**

Therefore, future clinical application would benefit from careful investigation of additional cytokine treatment to reduce chemotherapy-induced side effects.

## Background

Interleukin-6 (IL-6) is one of the best characterized cytokines and much is known about its pleiotropic behavior. As described by many groups, IL-6 is produced by both lymphoid and nonlymphoid cells, such as T cells, B cells, monocytes, endothelial cells, fibroblasts and many tumor cell types [1]. It is also therefore involved in many biological processes including upregulation of acute phase proteins during inflammation [2], immune regulation and hematopoiesis [3] as well as hemostasis which includes platelet formation [4]. Furthermore, IL-6 has been shown to play a pathogenic role in the development and progression of several tumor types [5]. In contrast to this, IL-6 has been shown to possess anti-tumor effects as in the example of treatment of B16 melanoma [6] or small cell lung carcinoma [7]. In both cases, the growth or proliferation inhibition could only be detected after addition of soluble IL-6 receptor (sIL-6-R) in cell culture systems, indicating that the pleiotropic effects of IL-6 may be due to differing mechanisms in signal transduction. Two different forms of the IL-6 receptor have been characterized which orchestrate the signaling pathways of the cytokine. IL-6 binds to either a specific receptor on the cell surface (mIL-6-R) of hepatocytes, monocytes/macrophages and leukocytes [8] or to the soluble, circulating sIL-6-R version generated by alternative splicing or by shedding of the mIL-6-R. The latter kind of signal transduction is named IL-6 trans-signaling (IL-6TS) which can potentiate the signal transduced by mIL-6R and also extend the range of target cells [9]. In both cases, the IL-6/(s/m)IL-6-R complex binds to the glycoprotein gp130 which then forms homodimers [10], leading to activation of the Ras/MAPK- and the JAK/STAT pathway [11]. The JAK/STAT pathway in turn results in phosphorylation of STAT3, followed by dimerization and accumulation in the cell nucleus where phosphorylated STAT3 dimers act as transcription factors [12].

To further characterize the IL-6TS, Rose-John et al. (1996) constructed a fusion protein, named hyper-IL-6, in which human IL-6 is covalently linked to the human sIL-6R [13]. The hyper-IL-6 designer cytokine activates cells in the same way as the IL-6/sIL-6-R complex [14]. However, its activity is highly enhanced (100-1000 fold) and the time of action is extended, which is probably due to its prolonged half-life in blood plasma [15].

Recombinant vaccinia virus strains (rVACV) not only feature pronounced oncolytic activity, but also serve as efficient vehicles for delivery and expression of proteins *in vivo*. The vaccinia virus vector system has the ability to stably integrate over 25,000 base pairs of foreign DNA into the viral genome and is further characterized by its cytoplasm-restricted gene expression [16]. In rVACV mediated oncolytic therapy, this can be used to insert diagnostic or therapeutic genes, including fluorescent proteins and luciferases [17], anti-angiogenic agents [18], or immunostimulatory cytokines such as GM-CSF [19].

In this study, we constructed the hyper-IL-6-encoding VACV strain GLV-1h90, which was derived from the previously described strain GLV-1h68 [20]. After systemic delivery, GLV-1h90 was capable of infecting and replicating in established DU-145 prostate xenograft tumors, leading to overexpression of hyper-IL-6 which in turn is secreted into the blood circulation. We used this model to analyze the effects of IL-6 that are exclusively transduced via the IL-6 trans-signaling pathway in combination with the oncolytic activity of the vaccinia virus, which included gain in body weight, levels of thrombocytosis and accelerated epidermal barrier repair.

We further report on a possible medical application for GLV-1h90. Recently, our group demonstrated that the combination of viral- and chemotherapy with a number of different chemotherapeutic agents (e.g. cisplatin or gemcitabine) improved the oncolytic activity of the recombinant vaccinia virus GLV-1h68 [21]. Unfortunately, chemotherapies often implicate a wide range of side effects especially due to non-specific involvement of all rapidly proliferating cells. One of the most common side effects is myelosuppression, which includes a decreased production of thrombocytes [22]. Chemotherapy-induced thrombocytopenia is a significant safety concern due to the increased risk of bleeding and can delay scheduled treatment [23]. For this reason, we used the hyper-IL-6-encoding VACV strain GLV-1h90 in combination therapy with the chemotherapeutic agent mitomycin C. This approach improves therapeutic outcomes and reduces negative side effects such as thrombocytopenia.

## Materials and methods

### Virus and cell culture

Androgen-insensitive DU-145 human prostatic cancer cells (authenticated by nonaplex PCR in 2011 [24]) were cultured in MEM containing 1% glutamaxx, 1% NEAA, 1% sodium pyruvate, 1,5 g/L sodium bicarbonate, 1% 100 × penicillin (10000 units/ml)/streptomycin (10 mg/mL) solution and 10% fetal bovine serum (FBS) (Invitrogen GmbH, Karlsruhe, Germany).

African green monkey kidney fibroblasts (CV-1) (ATCC-No. CCL-70) were maintained in Dulbecco's modified Eagle's medium (DMEM) supplemented with 1% 100 × penicillin (10000 units/mL)/streptomycin (10 mg/mL) solution and 10% FBS at 37°C under 5% CO_2_.

GLV-1h68 was derived from the vaccinia virus strain LIVP as described previously [20]. In GLV-1h90, the *gusA*-containing gene cassette was replaced by hyper-IL-6-encoding cDNA under control of a vaccinia virus synthetic early promoter (PE).

The plasmid DNA pCDM8-H-IL-6 for the construction of GLV-1h90 was kindly provided by Dr. Stefan Rose-John (University of Kiel, Germany), in which a fusion cDNA encoding a fusion protein of human IL-6 (corresponding to amino acid residues 29-212) fused to the C-terminus of human soluble receptor for IL-6 (sIL-6R, corresponding to amino acid residues 1-323) via a flexible peptide linker. This fusion cDNA was amplified with primers 5'-GTCGAC (*Sal I*) CCACCATGCTGGCCGTCGGCTGCGC-3' and 5'-GGTACC (*Kpn I*) CTAGAGTCGCG GCCGCGACC-3' in a PCR, and cloned into the pCR-Blunt II-TOPO vector (Invitrogen, Carlsbad, CA). The fusion cDNA was sequencing confirmed, released by *Sal I *and *Kpn I *digestion, and then subcloned into an intermediate vector, placing the cDNA under the control of a vaccinia synthetic early promoter (PsE). The vaccinia sIL-6R/IL-6 expression cassette was further subcloned into the vaccinia hemaglutinin (HA) transfer vector, yielding the final construct HA-SE-IL-6-1 that was used for recombination. GLV-1h90 was then generated by insertion of pSE-sIL-6R/IL-6 into the HA locus of parental strain GLV-1h68 via *in vivo *homologous recombination, interrupting the *gusA *expression cassette at the HA locus of GLV-1h68. For this, CV-1 cells were infected with GLV-1h68 at a multiplicity of infection (MOI) of 0.1 for 1 hour, then transfected with the hyper-IL-6 transfer vector HA-SE-IL-6-1 using Fugene (Roche, Indianapolis, IN, USA). Two days post infection (dpi), infected/transfected cells were harvested and the recombinant viruses were selected and plaque purified as described previously [25]. The genotype of hyper-IL-6 expressing GLV-1h90 was verified by PCR and sequencing. Also, the lack of expression of β-glucuronidase was confirmed by staining with 5-bromo-4-chloro-3-indolyl-β-D-glucuronic acid (X-GlcA; Research Product International, Mt. Prospect, IL, USA).

### Protein isolation and detection

At 2, 4, 8, 12, 24, 48, or 72 hours post-infection (hpi), DU-145 cells were harvested and lysed in RIPA buffer (25 mmol/L Tris-HCl pH 7.6, 150 mmol/L NaCl, 1% NP-40, 1% sodium deoxycholate, 0.1% SDS) supplemented with proteinase inhibitor cocktail (Roche, Penzberg, Germany). The cell lysates were separated on a 10% SDS-PAGE, and proteins were transferred onto a nitrocellulose membrane (Whatman GmbH, Dassel, Germany). The membrane was then incubated with anti-beta actin mouse monoclonal antibody (Abcam, Cambridge, UK), anti-beta galactosidase rabbit polyclonal antibody (Molecular Probes, Leiden, Netherlands), anti-GFP rabbit polyclonal antibody (Santa Cruz, Heidelberg, Germany), or anti-human IL-6 rat polyclonal antibody (BioLegend, San Diego, US) and detected using horseradish peroxidase (HRP) labeled secondary antibodies: anti-mouse (Abcam, Cambridge, UK), anti-rabbit (Abcam, Cambridge, UK) or anti-rat (Sigma-Aldrich, Taufkirchen, Germany), followed by enhanced chemiluminescence.

For the analysis of (Phospho-) STAT3 expression in DU-145 cells, the cells were incubated with sterile filtered (0.1 μm pore size) supernatants of mock-, GLV-1h68- or GLV-1h90-infected (MOI of 1.0 for 24 hours) DU-145 cells. Oncostatin M (Biomol GmbH, Hamburg, Germany), which also activates the JAK/STAT signaling cascade [26], was used as a positive control, while gp130/Fc chimera (R&D Systems, Minneapolis, MN, USA) were added into the supernatant of GLV-1h90-infected cells to prevent hyper-IL-6 from binding to the endogenous cellular gp130 receptors. Fifteen minutes later, cell lysates were prepared as described before, separated with a 10% SDS-PAGE and proteins were transferred onto a nitrocellulose transfer membrane. The membrane was then incubated with anti-STAT3 rabbit polyclonal antibody (Cell Signaling Technology, Danvers, MA) or anti-phospho-STAT3 rabbit polyclonal antibody (Cell Signaling Technology, Danvers, MA).

Quantitative IL-6 specific enzyme-linked immunosorbent assay (ELISA) was performed according to the manufacturer's protocols (R&D systems, Minneapolis, MN). DU-145 cell lysates and supernatants as well as tumor lysates and sera were analyzed at different time points. Absorbance was measured at 450 nm using a plate-reading spectrophotometer (SunriseTM, TECAN Group, Männedorf, Germany).

### Histology and fluorescent microscopy

For the microscopic analysis of (Phospho-) STAT3 expression and distribution, DU-145 cells were seeded on coverslips and incubated with sterile filtered (0.1 μm pore size) supernatant of mock-, GLV-1h68- or GLV-1h90-infected (MOI of 1.0 for 24 hours) DU-145 cells. Fifteen minutes later cells were fixed using 4% paraformaldehyde, washed in PBS and stained using anti-STAT3 rabbit polyclonal antibody (Cell Signaling Technology, Danvers, MA) or anti-phospho-STAT3 rabbit polyclonal antibody (Cell Signaling Technology, Danvers, MA) and Hoechst 33342 (Sigma-Aldrich, Taufkirchen, Germany). Microscopy was performed using a fluorescence microscope (Axiovert 200 M, Zeiss, Oberkochen, Germany) equipped with an AxioCam MR camera and the Axiovision 4.5 aquisition software. Digital images (1388 × 1040 pixel 16-bit grayscale images) were processed with Photoshop 7.0 (Adobe Systems, USA) and merged to yield pseudo-coloured pictures.

Histology and fluorescence microscopy of tumor sections were performed as described previously [27]. Tissue sections were stained using anti-human IL-6 rabbit polyclonal antibody (BioLegend, San Diego, US) and Hoechst 33342 (Sigma-Aldrich, Taufkirchen, Germany), respectively. After several rinses in PBS, tissue sections were mounted in Mowiol 4-88 (Sigma-Aldrich, Taufkirchen, Germany).

Tumor specimens were examined with the stereo-fluorescence microscope (MZ16 FA, Leica, Heerbrugg, Switzerland) equipped with a digital CCD camera (DC500, Leica) and the Leica IM1000 4.0 acquisition software. Digital images (1300 × 1030 pixel color images) were processed with Photoshop 7.0 (Adobe Systems, USA) and merged to overlay pictures.

For analysis of megakarypoiesis, semi-thin sections of the bone marrow were prepared. First femurs of mice were cut in ~0.5 cm pieces and fixed overnight at 4°C with 0.1 M sodium cacodylate (pH 7.2) (Roth, Karlsruhe, Germany) containing 2.5% glutaraldehyde (AppliChem, Darmstadt, Germany) and 2% formaldehyde (Sigma-Aldrich, Taufkirchen, Germany). The bone was removed with forceps before the remaining bone marrow was washed with 50 mM sodium cacodylate (pH 7.2) and subsequently fixed for 2 hours at 4°C with 2% osmium tetroxide (Roth, Karlsruhe, Germany) in 50 mM sodium cacodylate (pH 7.2). Samples were washed with distilled water and stained overnight with 0.5% aqueous uranyl acetate (Merck, Darmstadt, Germany), dehydrated with ethanol, and embedded in Epon 812 (Serva, Heidelberg, Germany). Two-hundred nm semi-thin sections were stained using 1% methylene blue (Merck, Darmstadt, Germany) in 1% sodium borate (Sigma-Aldrich, Taufkirchen, Germany) containing 1% Azure II (Merck, Darmstadt, Germany). For quantification of megakaryocytes 5-8 bone marrow sections from different layers of each sample (n = 4) were examined with an Axiovert 200 M microscope (Zeiss, Oberkochen, Germany) equipped with an AxioCam MRc 5 camera and the Axiovision 4.5 aquisition software. Small (~ 30 μm diameter) and large (~ 60 μm diameter) megakaryocytes were counted in randomly selected areas.

### Animal studies

DU-145 xenograft tumors were developed in 6-to-8-week-old female nude mice (NCI:Hsd:Athymic Nude-*Foxn1*^nu^, Harlan, Borchem, Germany and Indianapolis, USA) by implanting 5 × 10^6 ^DU-145 cells subcutaneously in the right hind flank. Fourteen days after tumor cell implantation, groups of five mice were injected with a single intravenous (i.v.) dose of PBS, or 5 × 10^6 ^plaque forming units (pfu) of GLV-1h68 or GLV-1h90 in 100 μL of PBS. Weights of the mice, number of tail lesions as well as tumor growth, in two dimensions using a digital caliper, were recorded twice a week. Tumor volume was calculated as ([length × width^2^] × 0.52) and reported in mm^3^. Concentration of blood platelets was counted 0, 5, 8, 11, 14, 16, 18, 21 and 25 dpi. All animal experiments were carried out in accordance with protocols approved by the Institutional Animal Care and Use Committee (IACUC) of Explora Biolabs (San Diego, USA, protocol number EB08-003) or the government of Unterfranken (Würzburg, Germany, protocol number AZ 55.2-2531.01-17/08).

### Tumor lysates

Tumors were collected 2, 5, 9 and 14 dpi, weighed, homogenized and added to 2x volume of lysis buffer (50 mM Tris-HCl with 2 mM EDTA, pH 7.4) supplemented with 2 mM phenylmethylsulfonyl fluoride and proteinase inhibitor cocktail (Roche Diagnostics GmbH, Penzberg, Germany). Samples were homogenized using a gentle MACS dissociator (Miltenyi Biotec) and centrifuged for 15 minutes (13000 g, 4°C).

### Platelet counting

Peripheral blood samples were obtained by retro-orbital plexus puncture using 20 μl heparinized capillary tubes (Hecht assistent, Sondheim, Germany). Mean platelet numbers were measured in 50 μL whole blood diluted with 50 μL ACD buffer (12 mM citric acid, 15 mM sodium citrate, 25 mM D-glucose) with a Sysmex KX-21 automatic micro-cell counter (Sysmex GmbH, Norderstedt, Germany).

### Combination therapy with mitomycin C

DU-145 tumor bearing mice with an average tumor volume of 250 mm^3 ^were divided into 3 groups (n = 10). One group was i.v. injected with a single dose of PBS, the second and third group with 5 × 10^6 ^pfu's of GLV-1h68 or GLV-1h90 in 100 μL of PBS. For combination therapy each group was subdivided into two groups which were injected with PBS or treated with i.v. injections of mitomycin C (Medac GmbH, Wedel, Germany) at 4 mg/kg/day on day 4 after PBS/virus injection and 3 mg/kg/day on day 5 after PBS/virus injection. Tumor volume, number of tail lesions as well as number of blood platelets was recorded over a time period of 25 dpi.

### Statistical analysis

A two-tailed Student's *t *test was used for statistical analysis. *P *values of ≤ 0.05 were considered statistically significant

## Results

### Recombinant gene expression in GLV-1h68- or GLV-1h90-infected DU-145 tumor cells

**Figure 1:**
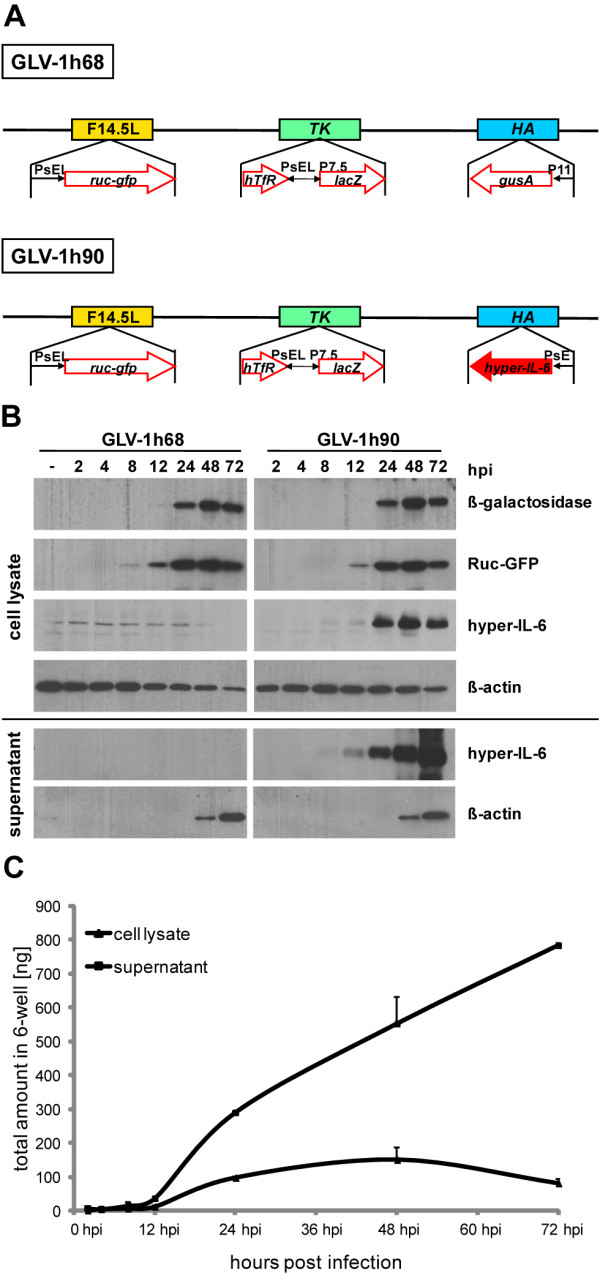
**Vaccinia virus strains and recombinant protein expression**. (A) Constructs of the recombinant vaccinia virus strains GLV-1h68 and GLV-1h90 which were derived from the wild-type Lister strain. The viral genes encoding for F14.5L, thymidine kinase (TK) and hemaglutinin (HA) were interrupted by the foreign gene expression cassettes encoding for a Renilla-luciferase-GFP (*Ruc-GFP*) fusion protein, beta-galactosidase (*lacZ*), beta-glucuronidase (*gusA*) and hyper-IL-6, respectively. (B) Infection of DU-145 cells led to expression of the marker genes as visualized by Western blot analysis of cell lysates and supernatants of infected cells at 2, 4, 8, 12, 24, 48, and 72 hpi. Mock-infected cells (-) served as negative control. (C) Quantitative (ELISA) measurements of (hyper-) IL-6 in cell lysates and supernatants of GLV-1h90-infected DU-145 cells.

Expression of the non-viral genes in the recombinant vaccinia virus strains used in this study was tested in the human prostatic cancer cell line DU-145. The cells were infected with the previously described GLV-1h68 [20] or the hyper-IL-6-encoding GLV-1h90 (Figure 1A).

Protein samples were obtained from infected DU-145 cells at different time points after infection with GLV-1h68 or GLV-1h90 at an MOI of 0.5. The expression of Ruc-GFP and beta-galactosidase was observed in cell lysates upon infection with either rVACV strains starting at 12 hpi. Concurrently, an increased amount of hyper-IL-6 was detectable in GLV-1h90-infected cells in contrast to GLV-1h68-infected cells, where only thin bands were observed indicating endogenously produced IL-6 (Figure 1B).

Since hyper-IL-6 must be secreted in order to bind the extracellular domain of gp130, the supernatant of infected cells was also analyzed for the presence of the fusion protein. Here, the secreted designer cytokine was detectable also at 12 hpi and the hyper-IL-6 concentration increased during the infection. This indicated active secretion of hyper-IL-6 by the infected tumor cells. At later stages, starting from 48 hpi, beta-actin was detectable in cell supernatants of GLV-1h68- or GLV-1h90-infected cells, indicating virus-mediated cell lysis (Figure 1B).

Increasing amounts of hyper-IL-6 in the supernatant of GLV-1h90-infected cells could also be confirmed using an IL-6-specific ELISA (Figure 1C) reaching a maximum of about 130 ng/mL at 72 hpi. On the other hand, no hyper-IL-6 was detectable in cell lysates or supernatants of GLV-1h68 or uninfected DU-145 cells (data not shown).

### Vaccinia virus-mediated hyper-IL-6 expression activates the JAK/STAT signaling pathway

IL-6 activates the JAK/STAT signaling pathway and leads to phosphorylation of Tyr-705 of the STAT3 protein [15]. Upon phosphorylation, STAT3 translocates into the nucleus ultimately leading to the expression of target genes involved in differentiation, survival, apoptosis and proliferation (reviewed in [28]).

To analyze the biological activity of GLV-1h90-encoded hyper-IL-6, the phosphorylation status of STAT3 and its translocation into the cell nucleus in DU-145 cells was determined after incubation with the supernatant of GLV-1h90-infected cells. DU-145 cells were treated with supernatants from mock-, GLV-1h68- and GLV-1h90-infected cells, respectively, and protein samples were harvested after 15 minutes. Oncostatin M, which also activates the JAK/STAT signal cascade [26], was used as a positive control, while gp130/Fc chimera was added in excess in supernatants of GLV-1h90-infected cells to prevent hyper-IL-6 from binding to the endogenous cellular gp130 receptors.

**Figure 2:**
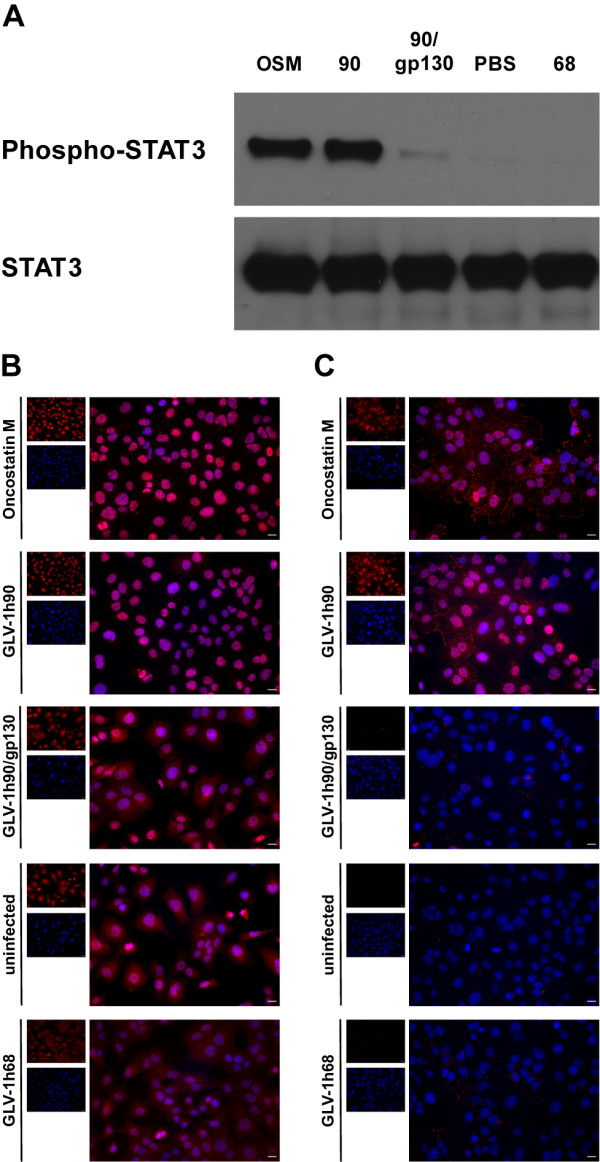
**Hyper-IL-6 dependent activation of the JAK/STAT signaling pathway**. (A) Western blot analysis of phosphorylated STAT3 (P-STAT3) and unphosphorylated STAT3 in DU-145 cells after co-incubation with sterile filtered supernatants of mock- (-), GLV-1h68- (68) or GLV-1h90- (90) infected cells. Oncostatin M (OSM) served as positive control and gp130/Fc chimera was added in excess to the supernatants of GLV-1h90-infected cells (90/gp130) to show specific activation via cellular gp130. Hyper-IL-6 and oncostatin M dependent migration of STAT3 into the nuclei (B) and phosphorylation (C) of STAT3 in DU-145 cells were proven by immunofluorescent microscopy. Overlay images (large picture) resulting from cells stained for (phosphorylated) STAT3 (red), and Hoechst 33342 stained nuclei (blue) were depicted. Scale bars indicate 8.6 μm.

As shown in Figure 2A STAT3 was exclusively phosphorylated in DU-145 cells treated with oncostatin M and the supernatant of GLV-1h90-infected cells (hyper-IL-6). The STAT3 tyrosin 705 phosphorylation induced by hyper-IL-6 could be blocked by addition of the gp130/Fc chimera, showing the specificity of hyper-IL-6 to gp130. Total STAT3 expression was not altered (Figure 2A lower panels) and used as loading control.

Immunofluorescence analysis of STAT3 (Figure 2B) and Phospho-STAT3 (Figure 2C) confirmed these results and showed activation of the JAK/STAT pathway. The anti-STAT3 antibody detected STAT3 regardless of its phosphorylation state. Therefore, it was possible to directly track the translocation of (Phospho-) STAT3 into the cells' nucleus of DU-145 cells incubated with the conditioned media of GLV-1h90-infected cells or oncostatin M. The (Phospho-) STAT3 protein was detected exclusively in the nucleus in DU-145 cells treated with the supernatant of GLV-1h90-infected cells or oncostatin M, whereas in the remaining experimental groups the STAT3 protein could be detected both in the cytoplasm and in the nucleus. Staining for Phospho-STAT3 confirmed this observation as Phospho-STAT3 could only be detected in the GLV-1h90 and the oncostatin M groups and was exclusively located in the cell nucleus.

Taken together, virally encoded hyper-IL-6 was efficiently and functionally secreted from infected tumor cells. In addition, these results clearly showed specific activation of the JAK/STAT pathway by vaccinia virus-encoded hyper-IL-6 in human prostatic cancer cells.

### Hyper-IL-6 expression could be detected both in tumors and in blood serum of GLV-1h90-injected mice

Similar to the observations made for GLV-1h68 [20], systemic administration of GLV-1h90 into tumor-bearing mice resulted in specific replication of vaccinia virus in tumor tissue (data not shown).

**Figure 3:**
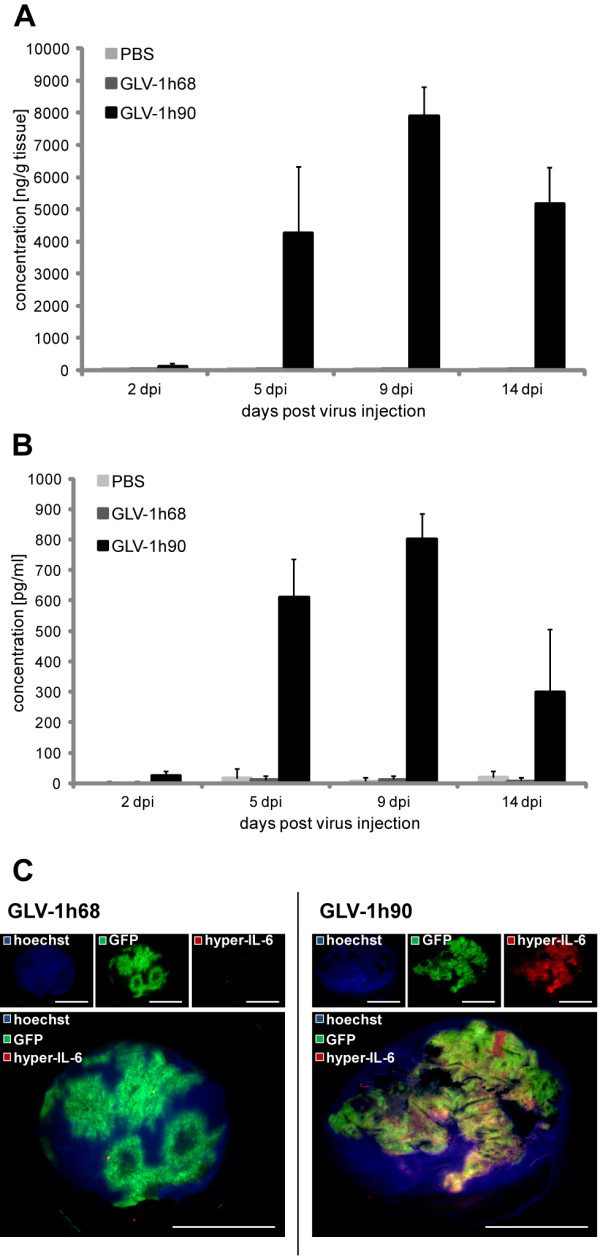
**Detection of hyper-IL-6 in tumors and blood serum samples of GLV-1h90-injected mice**. Quantification (ELISA) of hyper-IL-6 in tumor lysates (A) and blood serum samples (B) of mock-, 5 × 10^6 ^pfu GLV-1h68- or GLV-1h90-injected mice at 2, 5, 9 and 14 dpi. (C) Whole tumor cross sections (100 μm) 11 days post injection of 5 × 10^6 ^pfu GLV-1h68 or GLV-1h90 were stained with anti-IL-6 antibody (red) to confirm hyper-IL-6 expression only in GLV-1h90-infected tumors. Viral infection was indicated by GFP (green), and nuclei were stained with Hoechst 33342 (blue). Scale bars indicate 5 mm.

To demonstrate hyper-IL-6 expression in GLV-1h90-colonized tumors, tumors from PBS-, GLV-1h68-, or GLV-1h90-injected mice were isolated 2, 5, 9 and 14 dpi and tumor tissue homogenates were prepared. Quantitative protein analysis revealed high amounts of hyper-IL-6 in GLV-1h90-colonized tumors with a maximum concentration of about 7900 ng/g tissue at 9 dpi (Figure 3A). At this time point the concentration of IL-6 in control tumors of PBS- or GLV-1h68-injected mice was around 345-fold lower (on average 22,85 ng/g in PBS- and 23,45 ng/g in GLV-1h68-injected mice).

Of special interest was the presence of hyper-IL-6 in blood serum samples of GLV-1h90-injected mice (Figure 3B), which is essential for its ability to induce signal transduction pathways outside the tumor. Hyper-IL-6 was actively secreted into the blood circulation as indicated by the same detection pattern over infection time in both the tumor and the blood (Figure 3AB). The hyper-IL-6 concentration in the blood serum also reached its maximum 9 dpi and was ~ 800 pg/mL. In PBS-injected mice the IL-6 concentration was ~ 130-fold lower and ~ 65-fold lower in GLV-1h68-injected mice (on average 6 pg/mL and 12 pg/mL in PBS- and GLV-1h68-injected mice, respectively) (Figure 3B) compared to GLV-1h90-treated animals.

Also, fluorescence microscopy of tumor sections 11 dpi confirmed the expression of hyper-IL-6 (Figure 3C). While GFP could be observed in GLV-1h68- and GLV-1h90-infected tumors, high amounts of hyper-IL-6 could only be detected in GLV-1h90-colonized tumors. As expected, the distribution of the fusion protein correlated with the area of GFP expression, since both proteins were encoded by GLV-1h90.

### Effects of GLV-1h90 in a DU-145 xenograft mouse model

**Figure 4:**
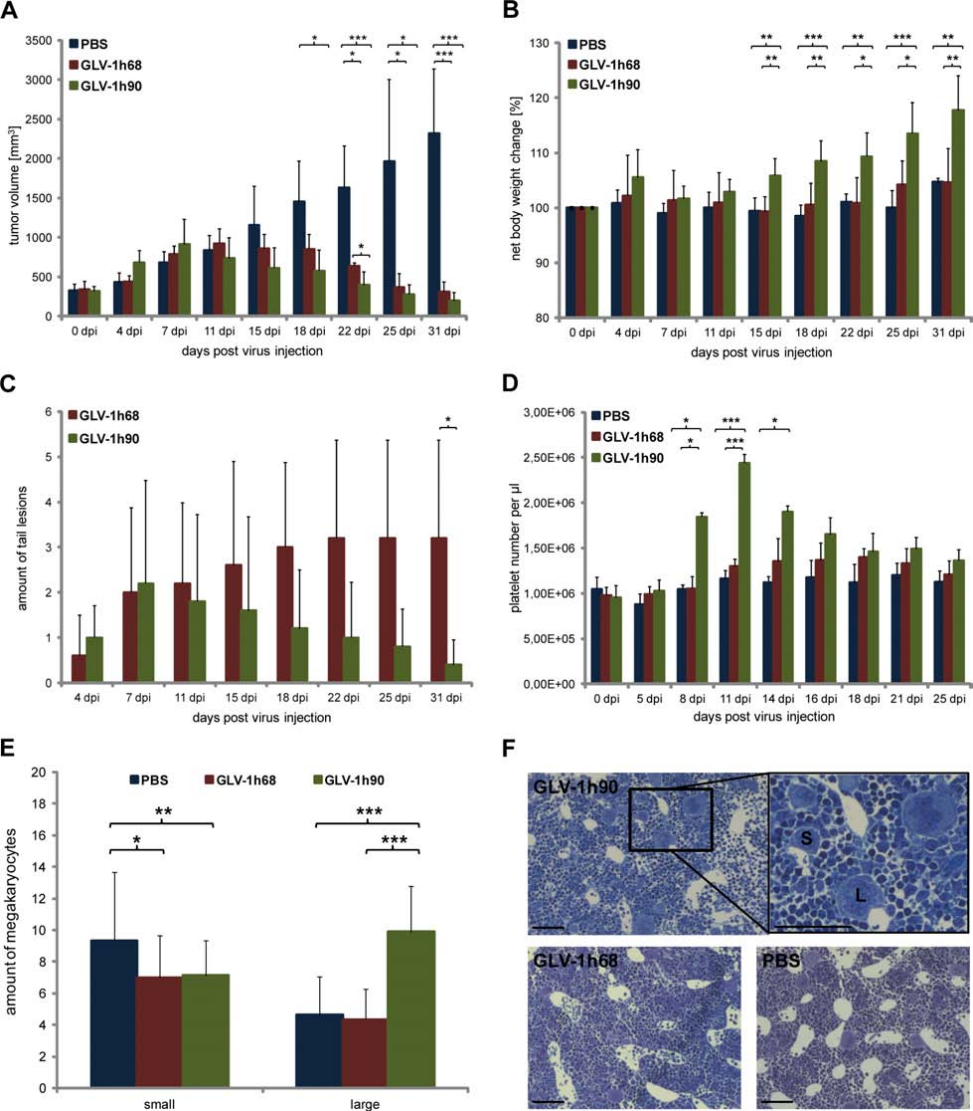
**Effects of GLV-1h90 in a DU-145 xenograft mouse model**. (A) Tumor measurements of DU-145 tumor bearing mice injected i.v. with PBS, 5 × 10^6 ^pfu GLV-1h68 or GLV-1h90 at 0, 4, 7, 11, 15, 18, 22, 25 and 31 dpi. (B) Net body weight change at the same time points. (C) Quantification of blood platelets in whole blood samples obtained by retro-orbital plexus puncture of PBS-, GLV-1h68- or GLV-1h90-injected mice. (D) Amount of tail lesions was counted over the whole experiment duration. Data represent average plus standard deviation for each group (n = 5). * indicates p ≤ 0.05; ** indicates p ≤ 0.01; *** indicates p ≤ 0.005. (E) Quantification of megakaryocytes of different maturation stages 7 dpi in semi-this sections of the bone marrow. Data represent average of 2 analyzed areas (20 × 22 cm) per section with 5-8 sections per sample (n = 4) plus standard deviation. * indicates p ≤ 0.05; ** indicates p ≤ 0.01; *** indicates p ≤ 0.005. (F) Examples for the analyzed bone marrow sections. S indicates small megakaryocytes; L indicates large megakaryocytes in advanced maturation phases; scale bars indicate 100 μm.

For analysis of oncolytic effects in live animals, DU-145 tumor-bearing mice were injected i.v. with either PBS or 5 × 10^6 ^pfu of GLV-1h68 and the hyper-IL-6-encoding GLV-1h90, respectively. Systemic administration of either viral constructs led to significant tumor regression starting 18 dpi compared to PBS injection. The comparison of both viral constructs revealed no significant differences in the inhibition of tumor growth except 22 dpi when GLV-1h90-colonized tumors were significantly smaller compared to those colonized with GLV-1h68 (Figure 4A), while viral titers in tumor tissue showed no relevant difference between both constructs (data not shown).

Besides the slightly increased oncolytic activity, GLV-1h90 revealed important benefits over GLV-1h68 mediated by the overexpression of the encoded designer cytokine hyper-IL-6. One of those advantages compared to GLV-1h68 concerned the healthiness of the mice during viral therapy. The health status of the mice was determined by measurement of the net body weight (body weight without tumor weight). As shown in Figure 4B, GLV-1h90 injection led to a significant increase in the net body weight starting 15 dpi compared to GLV-1h68 or PBS injection. However, it was not investigated whether the increased weight could be attributed to tissue or fluid, althogh no obvious fluid retentions were detected. Furthermore, mice injected with the hyper-IL-6-encoding VACV seemed to be more active, which indicated (together with the increased weight) an improved health status of the mice.

Another indication for the health status of the mice was the number and fate of tail lesions. Mice injected with either viral construct developed tail lesions at the site of virus injection starting 4 dpi which began increasing over time. Seven dpi, the number of GLV-1h90-induced tail lesions were reduced markedly and by 31 dpi a significant difference was observed compared to mice injected with GLV-1h68 (on average 0.4 in GLV-1h90- and 3.2 tail lesions in GLV-1h68-injected mice) (Figure 4C and Additional File 1 Figure S1). The observed accelerated barrier repair of tail lesions can be the result of direct or indirect antiviral activity mediated by IL-6 [29], keeping in mind that tail lesions are not only wounds which occur after tissue injury, but rather are associated with viral infection.

Barrier repair is a tightly regulated process in which blood platelets play an important role. Based on this, we analyzed whether overexpression of hyper-IL-6 leads to elevated levels of blood platelets in GLV-1h90-injected mice. Figure 4D shows that, in contrast to PBS or GLV-1h68, GLV-1h90 injection into DU-145 tumor-bearing mice significantly increased the number of platelets in the blood first detected 8 dpi and reached its maximum around 11 dpi which was about 2-fold increased compared to the other experimental groups. Subsequently, platelet concentration decreased and was nearly at baseline at 25 dpi. The number of neutrophils, lymphocytes and monocytes and red blood cells as well as hemoglobin levels showed normal levels with no differences between GLV-1h90-injected mice and the control groups (data not shown).

To further characterize if the increased platelet production was due to a hyper-IL-6-dependent stimulation of megakaryopoiesis in the bone marrow, megakaryocytes of different maturation phases were quantified in semi-thin cuts of the bone marrow. After injection of GLV-1h90, significant higher numbers of megakaryocytes could be detected in an advanced maturation phase (indicated by bigger size due to polyploidy) compared to GLV-1h68- or PBS-injected mice. At the same time, significant lower numbers of megakaryocytes could be visualized in early stages of megakaryopoiesis with smaller size in GLV-1h90-treated when compared to PBS-treated mice. The GLV-1h68-group showed lower megakaryocyte numbers in both analyzed maturation phases (Figure 4EF).

### Combination therapy with mitomycin C

**Figure 5:**
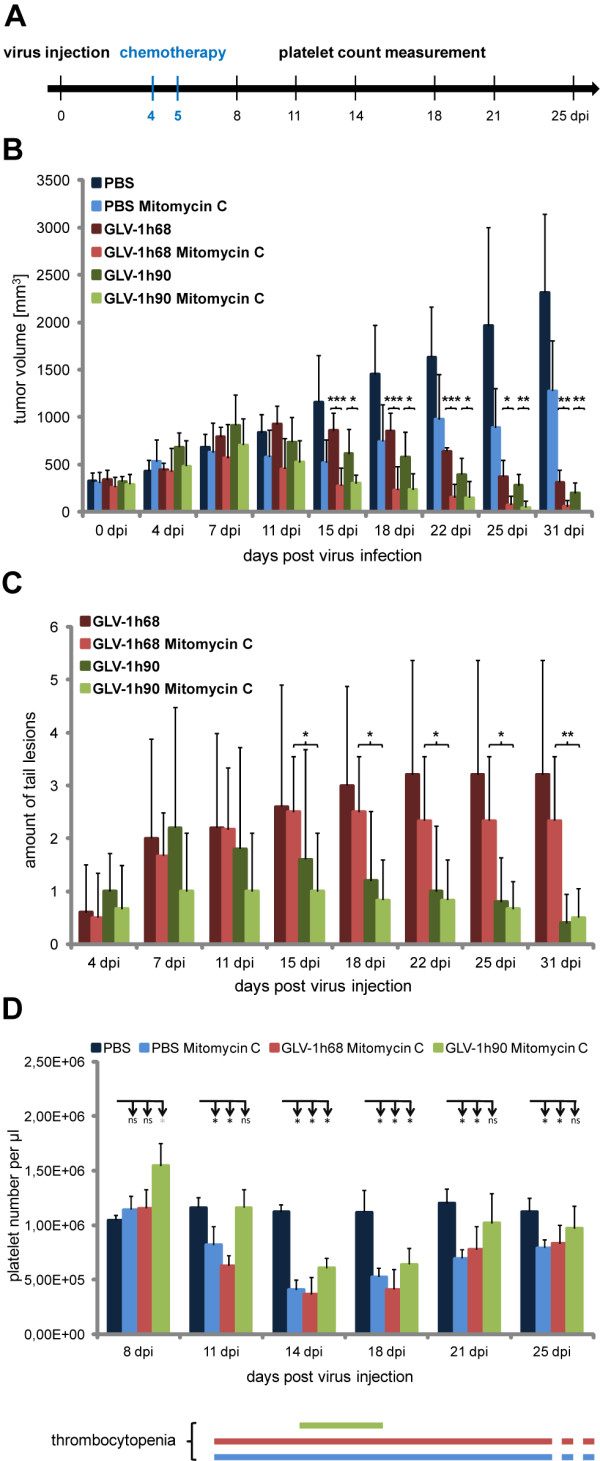
**Combination therapy of GLV-1h90 with mitomycin C**. (A) Injection schedule. (B) Tumor growth curve of DU-145 tumor bearing mice injected either with PBS, 5 × 10^6 ^pfu GLV-1h68 or GLV-1h90 alone or in combination with mitomycin C. Tumor volume was measured at 0, 4, 7, 11, 15, 18, 22 and 25 dpi. (C) Amount of tail lesions was counted starting 4 dpi, the time point when they occurred. (D) Quantification of blood platelets in whole blood samples of mice injected with PBS alone as well as of mice injected with PBS, GLV-1h68 or GLV-1h90 each in combination with mitomycin C at 8, 11, 14, 18, 21 and 25 dpi. The lower panel shows duration of thrombocytopenia in mice treated with mitomycin C. Thrombocytopenia is defined by a significantly decreased platelet number in relation to PBS-injected mice. Data in B, C and D represent average plus standard deviation for each group (n = 5). * indicates p ≤ 0.05; ** indicates p ≤ 0.01; *** indicates p ≤ 0.005; ns not significant.

Recently, we and others have demonstrated that the combination of oncolytic virotherapy with chemotherapy could lead to interactions that ultimately result in enhanced therapeutic effects [21,30]. On the other hand, chemotherapy is often associated with negative side effects, with thrombocytopenia one of the most frequently observed in cancer patients [31]. Consequently, we wanted to test the use of recombinant vaccinia virus-encoding hyper-IL-6 (GLV-1h90) instead of the parent virus GLV-1h68 in combination with the chemotherapeutic agent mitomycin C to improve not only the oncolytic effect but also the well being of the mice based on the observed effects of GLV-1h90. Mitomycin C is known to cause severe thrombocytopenia [32], which, as observed in first experiments, occurs around 7 days post chemotherapeutic treatment (data not shown). Therefore, as can be seen in the injection schedule (Figure 5A), we included mitomycin C injections at 4 and 5 days after initialization of virus treatment, assuming that GLV-1h90-induced production of thrombocytes could counteract chemotherapy-induced thrombocytopenia.

Combination therapy with mitomycin C and the oncolytic vaccinia virus strains led to a significantly improved DU-145 tumor regression compared to the respective monotherapies (Figure 5B). As early as 15 days post virus injection significant differences were observed between the groups injected with rVACV alone and those receiving the combination therapy regime. At that time, the tumor volume was already reduced to its initial dimension. In case of the rVACV monotherapy, it required ~ 22 days for the GLV-1h90- and 25 days for the GLV-1h68-injected group to reach the same tumor volume. Mitomycin C injection alone only led to tumor stagnation and therapeutic effects did not reach statistical significance when compared to PBS-treated controls.

As already observed in our previous experiment, tumor regression in GLV-1h90-injected animals was more pronounced compared to those injected with GLV-1h68, but again did not reach statistical significance. The same held true for the combination therapy with mitomycin C; although combination therapy was significantly better than the respective viral therapy alone, no significant differences in terms of tumor regression were observed between the GLV-1h68 and GLV-1h90 combination therapy groups.

However, analysis of the number of tail lesions revealed significant variations (Figure 5C and Additional File 2 Figure S2), indicating that combination of the viral constructs with the chemotherapeutic agent mitomycin C did not have any effects on wound healing. Approximately one week after virus injection the number of tail lesions decreased in GLV-1h90-injected mice while remaining stable or slightly increased in the GLV-1h68-injected groups.

Noteable was the finding that GLV-1h90 significantly reduced the time interval during which the mice suffered from thrombocytopenia (Figure 5D). Compared to PBS-injected mice, the treatment with mitomycin C resulted in significantly reduced platelet numbers for at least two weeks (from day 11 to at least day 25) in the PBS plus mitomycin C as well as in the GLV-1h68 plus mitomycin C groups. In contrast, combination of GLV-1h90 and mitomycin C led to a delayed occurrence of thrombocytopenia, as well as acceleration of the recovery process. Consequently, reduced platelet numbers were detectable for less than one week in the GLV-1h90 plus mitomycin C-injected group (14 to 18 dpi).

Taken together, functionally expressed hyper-IL-6 through i.v. delivered vaccinia virus optimized the combination therapy of the oncolytic virus GLV-1h90 and the chemotherapeutic agent mitomycin C. Besides the improved oncolytic effect, which also occurred with the parental strain GLV-1h68, GLV-1h90 was further able to significantly reduce thrombocytopenia, a negative side effect caused by the chemotherapeutic agent.

## Discussion

Among the currently investigated cancer therapies, the use of oncolytic vaccinia virus strains appear to be one of the most promising against a wide range of solid tumors [33]. While initial clinical trials using engineered oncolytic vaccinia virus strains have already been carried out [19,34], current research focuses on enhancing the already promising therapeutic effects of these viruses. This includes virus-mediated expression of immune-modulatory molecules [35,36], and combination with alternative therapies, such as prodrug therapy [37,38], radiation (Advani et al., submitted), or conventional chemotherapy [21,36]. The latter have been described to be very effective, but still suffer from the disadvantage of severe side effects.

Here, the hyper-IL-6-encoding rVACV strain GLV-1h90 was used to reduce the side effects resulting from the chemotherapeutic agent mitomycin C. This chemotherapeutic agent, as well as most other agents, is known to cause severe thrombocytopenia in mice as well as in human patients [32,39]. Interleukin-6 is a cytokine with multifunctional actions including regulation of immune responses [40,41], induction of acute-phase reactant release from hepatocytes [42] and enhancement of proliferation of hematopoietic progenitors [3]. In particular, the IL-6-promoted megakaryocyte maturation in the absence of other added growth factors, which leads to elevated platelet production [43,44] is an important focus when contemplating its implications on chemotherapy-induced thrombocytopenia.

Systemic administration of GLV-1h90 into DU-145 tumor-bearing mice led to significant tumor regression compared to mock-injected mice. This anti-tumor effect in this tumor model, as well as others, has been shown to be similar to that achieved by treatment with GLV-1h68 (data not shown). This result suggests that hyper-IL-6 itself has no significant direct anti-tumor activity or that virus-mediated oncolysis is, in the model systems tested, the most prominent factor responsible for tumor destruction, which may therefore mask potential cytokine effects.

However, our results clearly indicated that hyper-IL-6 maintained its biological activity when encoded by the oncolytic vaccinia virus strain. Large amounts of the cytokine were produced in and secreted from infected tumor cells in cell culture as well as in live animals. Secretion is crucial since the presence of hyper-IL-6 in blood is needed for the designer-cytokine to act outside the tumor tissue such as on megakaryocytes in the bone marrow [43,44] or on skin keratinocytes [45].

Hyper-IL-6-dependent activation of the JAK/STAT pathway was demonstrated on a molecular level by Western blot analysis and immunohistochemistry in cell culture. Furthermore, functionality of hyper-IL-6 in tumor-bearing mice resulted in elevated acute phase proteins (data not shown) as well as in accelerated epithelial barrier repair of tail lesions. IL-6 is known to affect multiple processes which are related to wound healing including attraction of neutrophils and their adhesion to dermal fibroblasts [46], recruitment of monocyte/macrophages responsible for clearance of debris and the provision of growth and angiogenic factors [47] and stimulation of keratinocyte proliferation which accelerates re-epithelialization after injury [45,48]. Furthermore, the observed accelerated barrier repair of tail lesions can be the result of direct or indirect antiviral activity mediated by IL-6 [29], keeping in mind that tail lesions are not only wounds which occur after tissue injury, but rather are associated with viral infection. Thus, future experiments are planned to investigate which processes are crucial in the here observed improved wound healing.

In addition, elevated platelet numbers in the blood circulation of mice injected with the hyper-IL-6 virus may also contribute to the accelerated healing of tail lesions [49]. In the blood of GLV-1h90-injected mice the platelet concentration increased after 5 dpi and reached its maximum around 11 dpi after which their concentration declined. A similar peak, about 2-3 days earlier, was also observed for the hyper-IL-6 concentration in the tumors as well as in the sera of GLV-1h90-injected mice. This suggests that, as a result of the intratumoral overexpression, hyper-IL-6 ends up in the blood circulation and can reach the bone marrow, where the designer cytokine can stimulate megakaryopoiesis. This process in turn leads to increased production of platelets, which could then be found in the blood several days later [43]. This hypothesis could be proven by quantification of different maturation phases of megakaryocytes in semi-thin cuts of the bone marrow. Besides thrombopoietin (TPO), the main regulator of megakaryopoiesis, many pro-inflammatory cytokines are known to stimulate, either alone or in conjunction with TPO, platelet production during megakaryopoiesis. While interleukin-3 is essential in early steps of megakaryopoiesis, IL-6 is postulated to be mainly involved in a process called endomitosis, which include megakaryocyte proliferation, cell growth as well as formation of polyploidy at the latest phase of megakaryopoiesis. The observation that, compared to both control groups, 7 dpi significant higher numbers of megakaryocytes in advanced maturation phases could be detected in bone marrow sections of GLV-1h90-injected mice confirms the assumption that hyper-IL-6 was able to stimulate megakaryopoiesis which in turn leads to elevated platelet numbers 3-4 days later. The platelet decrease might reflect the therapeutic effects caused by the virus, reducing the amount of viable (hyper-IL-6 producing) tumor cells. The results also illustrate that possible complications associated with the overexpression of cytokines can be self-limiting due to the destruction of the virus replication site.

Here, we showed for the first time, that oncolytic tumor therapy could be optimized through reduction of therapeutic side effects associated with chemotherapy such as thrombocytopenia, in mice receiving combination therapy with mitomycin C by using a hyper-IL-6-encoding vaccinia virus. The time period that mice experienced thrombocytopenia was reduced by at least 50% (less than one week compared to at least 2 weeks). Moreover, the combination of mitomycin C with either of the oncolytic vaccinia virus strains GLV-1h68 or GLV-1h90 resulted in significantly enhanced therapeutic effects.

Taken together, GLV-1h90 resulted in the production of the functionally active designer-cytokine hyper-IL-6 thereby retaining the oncolytic effects of its parental strain GLV-1h68. Maybe even more importantly, GLV-1h90 significantly improved the wellbeing of mice during combination therapy with mitomycin C.

For translation into the clinic, future studies on a higher number of tumor models will have to show if this also holds true for combinations with other chemotherapeutic agents, and whether other cytokines such as IL-2 and IL-11 that are already applied for treatment of thrombocytopenia in cancer patients receiving chemotherapy, will be as efficacious or maybe even superior to hyper-IL-6 in reducing thrombocytopenia and improving overall wellbeing in combination with oncolytic viral therapy.

## Conclusion

We have shown that the oncolytic vaccinia virus strain GLV-1h90 can be used to functionally express the designer cytokine hyper-IL-6 in human tumor xenografts of live mice. Hyper-IL-6 was secreted from the infected tumor tissue into the circulatory system enabling it to induce systemic effects. This was used, for the first time, to improve combination of oncolytic vaccinia virus therapy with conventional chemotherapy. Although this has previously been shown to have remarkable success in preclinical tumor models, the virus-mediated expression of the designer cytokine hyper-IL-6 offered a new approach to significantly reduce the duration of thrombocytopenia, a serious side effect of chemotherapy. Therefore, future pre-clinical studies investigating combination treatment with chemotherapy would benefit from careful investigation into the possibility of additional cytokine expression to reduce chemotherapy-induced side effects before progression to clinical trials.

## List of abbreviations

dpi: days post infection; ELISA: enzyme-linked immunosorbent assay; FBS: fetal bovine serum; GFP: Green fluorescent protein; GM-CSF: *Granulocyte-macrophage colony-stimulating factor*, hpi: hours post-infection; HA: hemagluttinin; HRP: horseradish peroxidase; IL: interleukin; IL-6TS: IL-6 trans-signaling; i.v.: intravenous; mIL-6-R: membrane bound IL-6 receptor; rVACV: recombinant vaccinia virus strain; sIL-6-R: soluble IL-6 receptor; TK: thymidine kinase.

## Competing interests

The research was supported by the Research and Development Division of Genelux Corp., San Diego, USA, and a Service Grant to the University of Würzburg, Germany also funded by Genelux Corp., San Diego, USA. JS, NGC, QZ and AAS are employees and shareholders of Genelux Corporation and Genelux GmbH respectively. SW, UD, GK, CR and SG are employees of the University of Würzburg. JBS and MH are supported by graduate stipends from Genelux Corporation. The funders had no role in study design, data collection and analysis or decision to publish. No competing intersts exist for JBS, MH, SW, UD, GK, CR and SG.

## Authors' contributions

JBS conceived the study, designed, performed and analyzed all experiments and wrote the manuscript. MH and UD participated in experimental design and performance. JS participated in conceiving the study, experiment analyzation and manuscript writing. SW, GK, CR and SG participated in conceiving the study. NGC, QZ and YAY participated in manuscript writing and provided essential material. AAS participated in conceiving the study and writing the manuscript. All authors read and approved the final version of the manuscript.

## Authors' information

JS, NGC, QZ and AAS are employees and shareholders of Genelux Corporation and Genelux GmbH respectively. SW, UD, GK, CR and SG are employees of the University of Würzburg. JBS and MH are supported by graduate stipends from Genelux Corporation. The funders had no role in study design, data collection and analysis or decision to publish.

## Supplementary Material

Additional file 1 **Figure S1. Effect of hyper-IL-6 on epithelial barrier repair of tail lesions**. Fluorescence microscopy of tail lesions of DU-145 tumor-bearing mice injected with 5 × 10^6 ^pfu GLV-1h68 (left panels) or GLV-1h90 (right panels), respectively. Viral infection was indicated by GFP (green). Images were taken at 25 dpi with a stereo-fluorescence microscope (MZ16 FA, Leica, Heerbrugg, Switzerland) equipped with a digital CCD camera (DC500, Leica) and the Leica IM1000 4.0 acquisition software. Digital images (1300 × 1030 pixel color images) were processed with Photoshop 7.0 (Adobe Systems, USA) and merged to overlay pictures. Scale bars in the upper panels indicate 5 mm; scale bars in the lower panels indicate 1 mm. Click here for file

Additional file 2 **Figure S2. Epithelial barrier repair of GLV-1h68- or GLV-1h90-induced tail lesions in combination therapy with mitomycin C**. Fluorescence microscopy of tail lesions of DU-145 tumor bearing mice injected either with 5 × 10^6 ^pfu GLV-1h68 or GLV-1h90 alone or in each case in combination with mitomycin C at 25 dpi. Viral infection was indicated by GFP (green). Images were taken with a stereo-fluorescence microscope (MZ16 FA, Leica, Heerbrugg, Switzerland) equipped with a digital CCD camera (DC500, Leica) and the Leica IM1000 4.0 acquisition software. Digital images (1300 × 1030 pixel color images) were processed with Photoshop 7.0 (Adobe Systems, USA) and merged to overlay pictures. Scale bars indicate 5 mm. Click here for file
