# Supplementary figures and images for: Functional hyper-IL-6 from vaccinia virus-colonized tumors triggers platelet formation and helps to alleviate toxicity of mitomycin C enhanced virus therapy
Source: J Transl Med. 2012 Jan 11;10:9. doi: 10.1186/1479-5876-10-9 (PMC3268093; doi:10.1186/1479-5876-10-9)

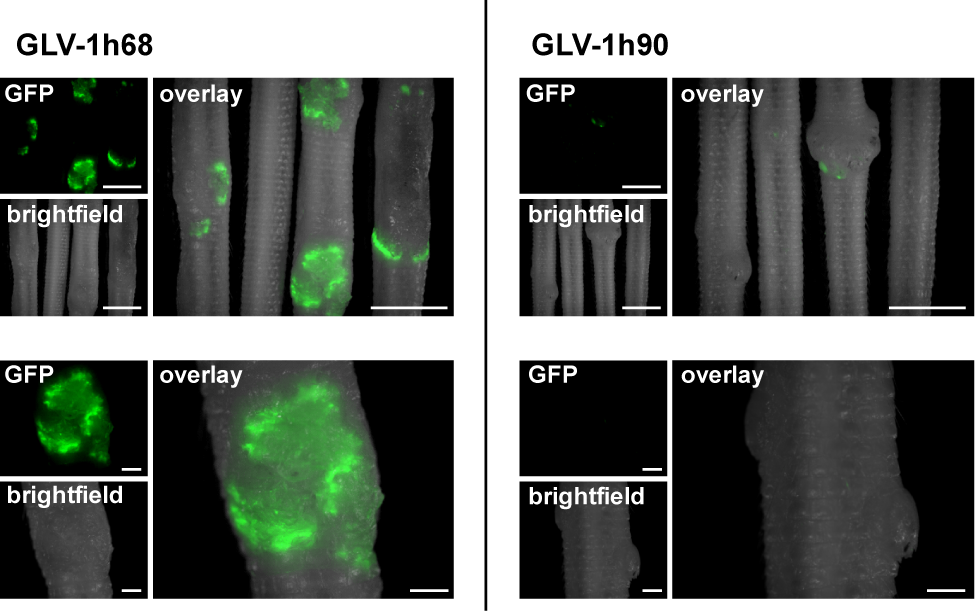

Supplement: Additional file 1 — Figure S1. Effect of hyper-IL-6 on epithelial barrier repair of tail lesions. Fluorescence microscopy of tail lesions of DU-145 tumor-bearing mice injected with 5 × 10^6^ pfu GLV-1h68 (left panels) or GLV-1h90 (right panels), respectively. Viral infection was indicated by GFP (green). Images were taken at 25 dpi with a stereo-fluorescence microscope (MZ16 FA, Leica, Heerbrugg, Switzerland) equipped with a digital CCD camera (DC500, Leica) and the Leica IM1000 4.0 acquisition software. Digital images (1300 × 1030 pixel color images) were processed with Photoshop 7.0 (Adobe Systems, USA) and merged to overlay pictures. Scale bars in the upper panels indicate 5 mm; scale bars in the lower panels indicate 1 mm. [file 1479-5876-10-9-S1.TIFF]

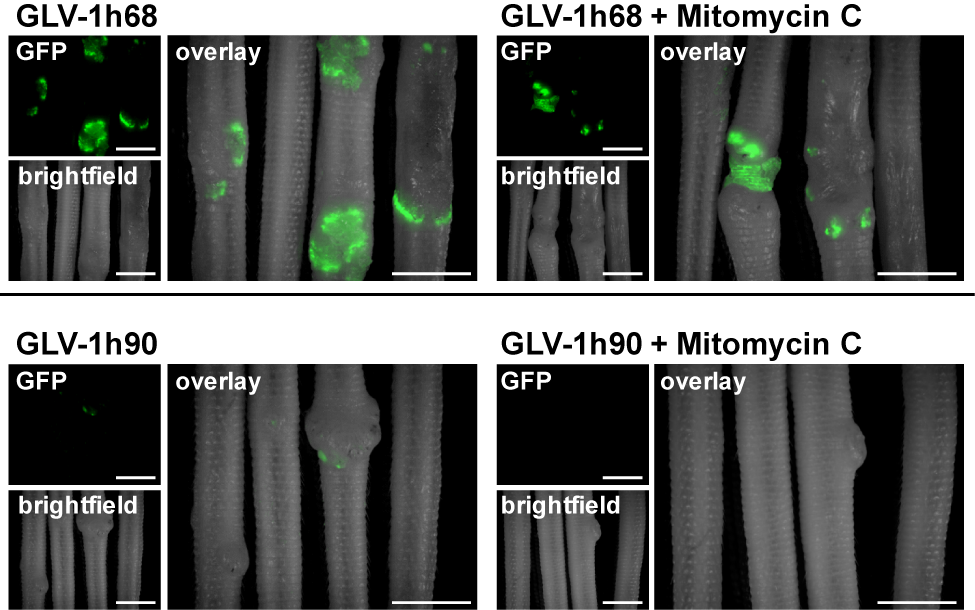

Supplement: Additional file 2 — Figure S2. Epithelial barrier repair of GLV-1h68- or GLV-1h90-induced tail lesions in combination therapy with mitomycin C. Fluorescence microscopy of tail lesions of DU-145 tumor bearing mice injected either with 5 × 10^6^ pfu GLV-1h68 or GLV-1h90 alone or in each case in combination with mitomycin C at 25 dpi. Viral infection was indicated by GFP (green). Images were taken with a stereo-fluorescence microscope (MZ16 FA, Leica, Heerbrugg, Switzerland) equipped with a digital CCD camera (DC500, Leica) and the Leica IM1000 4.0 acquisition software. Digital images (1300 × 1030 pixel color images) were processed with Photoshop 7.0 (Adobe Systems, USA) and merged to overlay pictures. Scale bars indicate 5 mm. [file 1479-5876-10-9-S2.TIFF]
